# Post-marketing surveillance of quizartinib for relapsed or refractory *FLT3*-ITD-positive acute myeloid leukemia in Japan

**DOI:** 10.1007/s12185-026-04181-7

**Published:** 2026-02-26

**Authors:** Yasushi Miyazaki, Itaru Matsumura, Takeshi Arita, Ryouko Fukuda, Mie Yamanaka

**Affiliations:** 1 Department of Hematology, Atomic Bomb Disease Institute, Nagasaki University, Nagasaki, Japan; 2 Department of Hematology and Rheumatology, Faculty of Medicine, Kindai University, Osaka-Sayama, Japan; 3 Division of Cardiovascular Medicine, Fukuoka Wajiro Hospital, Fukuoka, Japan; 4 Daiichi Sankyo Co., Ltd., 3-5-1 Nihonbashi-honcho, Chuo-ku, Tokyo, 103-8426 Japan

**Keywords:** Acute myeloid leukemia, Japan, Post-marketing surveillance, QT interval prolongation, Quizartinib

## Abstract

Quizartinib is an oral, once-daily, highly potent and selective second-generation, type II FMS-like tyrosine kinase 3 (FLT3) inhibitor, approved for the treatment of newly diagnosed and relapsed/refractory (R/R) *FLT3*-internal tandem duplication (ITD)-positive acute myeloid leukemia (AML). This descriptive, observational post-marketing surveillance (PMS) investigated the safety of quizartinib in patients with R/R *FLT3*-ITD-positive AML who initiated quizartinib between October 2019 and December 2021 in Japan. The safety analysis dataset included 126 patients (mean age 60.4 years) who consented to data publication. Eighty patients (63.5%) had previously received another FLT3 inhibitor. The median treatment duration was 71.0 days. Adverse drug reactions (ADRs) occurred in 68 patients (54.0%); grade ≥ 3 and serious ADRs occurred in 44 (34.9%) and 20 (15.9%) patients, respectively. QT interval prolongation occurred in 18/124 patients (14.5%), and the first event typically occurred < 30 days, but sometimes ≥ 150 days, after quizartinib initiation; none of these patients experienced clinical symptoms associated with the ADR. This PMS showed that the safety profile of quizartinib in routine clinical practice in Japanese patients with R/R *FLT3*-ITD-positive AML did not differ from the known safety profile of quizartinib. QT interval prolongation was manageable with current risk minimization measures.

## Introduction

Approximately 25% of patients with acute myeloid leukemia (AML) have internal tandem duplication (ITD) mutations in the gene for FMS-like tyrosine kinase 3 (*FLT3*) [1]. *FLT3*-ITD is prognostic for AML with a high frequency of relapse and reduced response to salvage therapy, and shorter overall survival (OS), compared with *FLT3* wild-type disease [2–4].

Quizartinib is an oral, once-daily, highly potent and selective second-generation, type II FLT3 inhibitor [5, 6]. Based on the QuANTUM-First study [7], quizartinib was approved in the USA [8, 9], European Union [10, 11], United Kingdom [12, 13], and Japan [14], in combination with standard induction and consolidation chemotherapy, followed by single agent maintenance therapy (but not after transplantation in the USA), for the treatment of adult patients with newly diagnosed *FLT3*-ITD-positive AML. In the global phase 3 QuANTUM-R study in patients with relapsed/refractory (R/R) *FLT3-*ITD-positive AML, quizartinib monotherapy improved OS compared with salvage chemotherapy (selected by the investigator before randomization as either mitoxantrone, etoposide, and cytarabine; fludarabine, cytarabine, and granulocyte colony-stimulating factor with idarubicin; or low-dose cytarabine) [6]. Based on the results of QuANTUM-R [6], quizartinib was approved in Japan for the treatment of patients with R/R *FLT3*-ITD-positive AML [15, 16].

Quizartinib and its active metabolite inhibit both the human ether-a-go-go-related gene (hERG) current and the slowly activating component of delayed rectifier potassium currents (IKs) [17]. Non-clinical studies demonstrated inhibition of the IKs channel by quizartinib at clinically relevant concentrations. Dose-dependent and concentration-dependent QT interval prolongation is an important adverse event (AE) associated with quizartinib treatment [17]. In phase 1 and 2 studies, the dose-limiting toxicity of quizartinib was QT interval prolongation on electrocardiogram (ECG), as measured by the QT interval corrected according to the Fridericia formula (QTcF) [18, 19]. In the QuANTUM-R study, the mean increase from baseline in QTcF at the maximum dose of quizartinib (60 mg/day) was estimated to be 21.1 ms [17].

Due to limited safety information on the use of quizartinib in the Japanese population, post-marketing surveillance (PMS) of all patients initiating quizartinib (up to a target sample size) to assess its safety in routine clinical practice in Japan was required as an approval condition of quizartinib. Here, we present the first report on the results of this PMS, particularly regarding QT interval prolongation associated with the use of quizartinib in patients with R/R *FLT3*-ITD-positive AML.

## Materials and methods

### Design and patients

The objective of this descriptive, observational, all-case PMS (jRCT1080224910) was to assess the occurrence of specific safety endpoints in Japanese patients treated with quizartinib for R/R *FLT3*-ITD-positive AML, and to collect information on other safety endpoints and treatment effectiveness. The PMS included all patients with R/R *FLT3*-ITD-positive AML who initiated treatment with quizartinib in Japan between October 10, 2019 and December 31, 2021. In Japan, the initial dose of quizartinib for adult patients with R/R *FLT3*-ITD-positive AML is 26.5 mg orally once daily for 2 weeks, and then increased to 53 mg orally once daily thereafter [16]. The dose is reduced by one level (to 26.5 mg once daily) when co-administered with a strong cytochrome P450 (CYP) 3A inhibitor and may also be reduced by one level according to the patient’s condition [16].

Patient data were collected from October 10, 2019 to June 30, 2022 (survey period) using a central registration method. Patients were observed for 6 months from the start of quizartinib treatment (i.e., the observation period), or until the day on which quizartinib was discontinued during the observation period (whichever occurred first).

The protocol of this PMS was reviewed by the Pharmaceuticals and Medical Devices Agency in Japan and the PMS was conducted in accordance with the Japanese Good Post-marketing Study Practice regulations. Based on these regulations, consent to participate in the PMS was not required. Written consent was obtained from patients (including proxies) enrolled in the PMS and participating institutions for publication of the results. Only patients who consented to publication of their data were included in the safety analysis dataset.

### Assessments

#### Safety

All treatment-emergent AEs (TEAEs) that occurred during the observation period (and the following day) were investigated. The severity of AEs was assessed by the investigator based on Common Terminology Criteria for Adverse Events version 5.0 from the Japanese Cooperative Oncology Group (CTCAE v5.0 JCOG). AEs for which a causal relationship to quizartinib could not be ruled out by the investigator were defined as adverse drug reactions (ADRs). Serious TEAEs or serious ADRs were defined as events that resulted in any of the following: death; a life-threatening condition; hospitalization or prolonged hospitalization; permanent or significant disability or dysfunction; a congenital anomaly; or any other medically important event.

The safety specification AEs of this PMS were QT interval prolongation, myocardial infarction, acute kidney injury, interstitial lung disease, and differentiation syndrome. The safety specification AEs were identified based on the following definitions using the Medical Dictionary for Regulatory Activities Japanese Version (MedDRA/J) version 25.1: QT interval prolongation (Standardized MedDRA Query [SMQ]: Torsade de pointes/QT prolongation [broad]); myocardial infarction (SMQ: myocardial infarction [broad]); acute kidney injury (preferred term: acute kidney injury); interstitial lung disease (SMQ: interstitial lung disease [narrow]); differentiation syndrome (events reported by the investigator as ‘events related to differentiation syndrome-like symptoms’ [these were defined as dyspnea, pyrexia, cough, peripheral edema, hypotension, weight gain, acute respiratory distress, pulmonary infiltration, pericardial effusion, acute kidney injury, musculoskeletal pain, hyperbilirubinemia, pulmonary hemorrhage, acute febrile neutrophilic dermatosis, and other possible differentiation syndrome-like symptoms commonly observed in acute promyelocytic leukemia differentiation syndrome]). QT interval, corrected as QTcF, was assessed by ECG measurements at baseline, week 1, and week 2 (before dose escalation), 1 week after dose escalation, 2 weeks after dose escalation, then monthly thereafter.

#### Effectiveness

Effectiveness was assessed by the physician. The best antitumor response was assessed by the investigator based on bone marrow blast count and peripheral blood neutrophil and platelet counts. The best antitumor response was categorized as complete remission (CR), CR without platelet recovery (CRp), or CR without neutrophil recovery (CRi), according to the antitumor efficacy criteria of a Japanese phase 2 study of quizartinib in patients with R/R *FLT3*-ITD-positive AML [20]. The composite CR (CRc) rate was defined as the proportion of patients who achieved CR, CRp, or CRi.

The PMS also investigated whether allogeneic hematopoietic stem cell transplantation (HSCT) was performed after quizartinib treatment without other AML therapies.

### Statistical analysis

The target sample size was 210 patients for this analysis, as 209 patients were estimated to provide a ≥ 90% probability of detecting at least one patient with interstitial lung disease, which of the safety specifications in this PMS had the lowest incidence in previous studies [6, 20]. Descriptive statistics were used in this analysis, with continuous values reported as means, standard deviations (SDs), medians, and ranges and categorical values reported as number and proportions. Point estimates and 95% confidence intervals (CI) using Clopper–Pearson’s CI were estimated for items related to safety and effectiveness. A subgroup analysis was also conducted to evaluate effectiveness by sex, age (< 65 vs ≥ 65 years), Eastern Cooperative Oncology Group performance status (ECOG PS; ≤ 2 vs ≥ 3), AML mode of onset (de novo vs secondary), central nervous system involvement (no vs yes), and previous use of other FLT3 inhibitors (no vs yes). Statistical analysis was conducted using SAS® basic version 9.4 (Cary, NC, USA).

## Results

### Patient disposition and characteristics

**Fig. 1:**
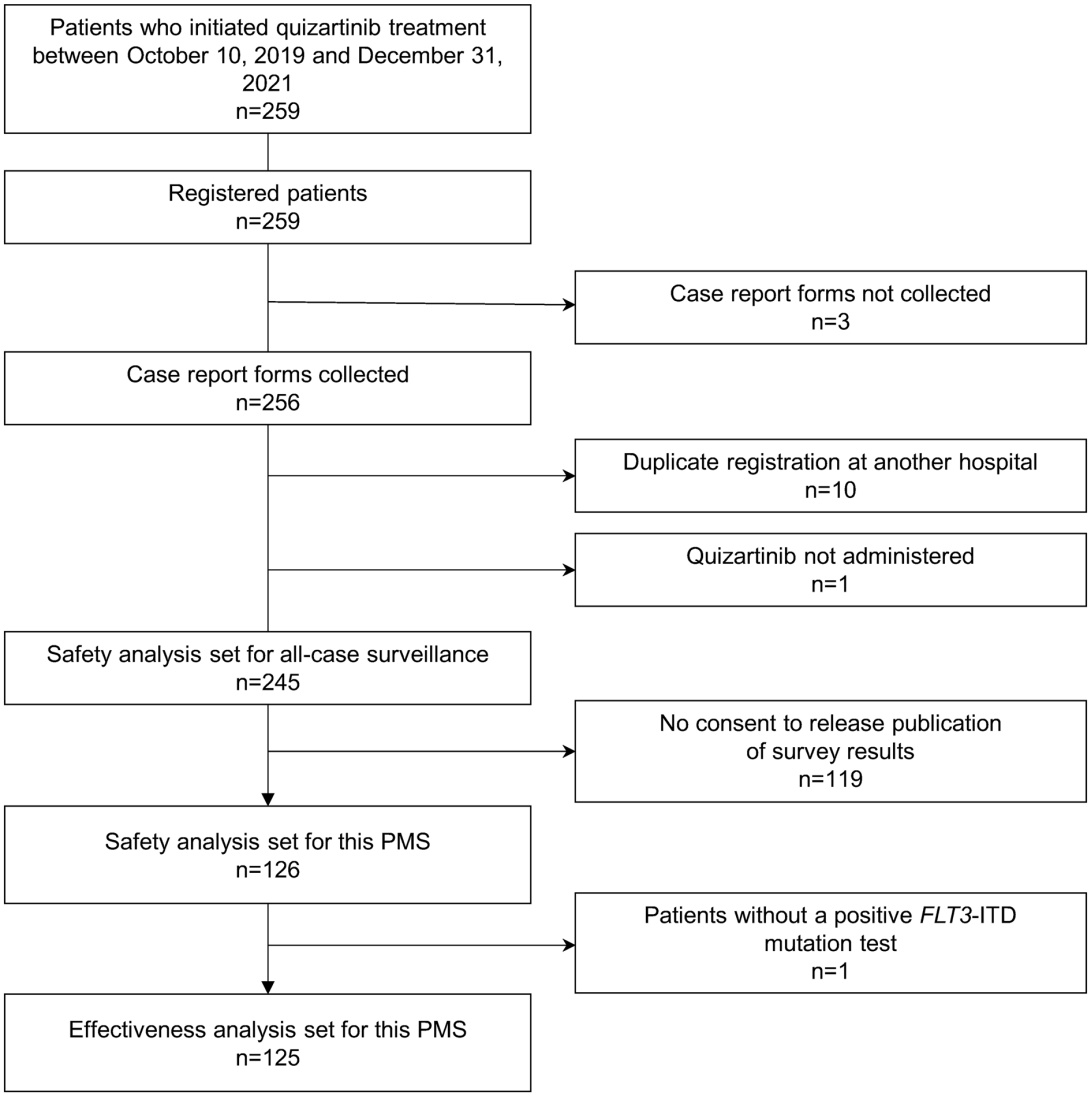
Patient disposition. *FLT3*-ITD, FMS-like tyrosine kinase 3 with an internal tandem duplication; PMS, post-marketing surveillance

Between October 10, 2019 and December 31, 2021, 259 patients with R/R *FLT3*-ITD-positive AML were treated with quizartinib at 229 institutions in Japan (Fig. 1). Case report forms were collected from 256 patients, of whom 10 were duplicates (double-registered at different institutions). Once duplicates were removed, and one patient who did not receive quizartinib was excluded, the overall safety analysis population included 245 patients. Consent for publication of results from this PMS was obtained from 126/245 patients, who comprised the safety analysis dataset for this PMS. For the effectiveness evaluation, one patient was excluded due to discontinuing quizartinib without a positive *FLT3*-ITD mutation test after starting treatment; diagnostic results were only available after quizartinib administration began. Therefore, effectiveness was evaluated in 125 patients.

The mean ± SD age of the safety analysis dataset at the start of quizartinib administration was 60.4 ± 17.7 years (Table 1). A noteworthy point is that 80 patients (63.5%) had previously been treated with another FLT3 inhibitor. Of these, 52 patients (65.0%) discontinued the previous FLT3 inhibitor due to resistance and switched to quizartinib.

**Table 1:** Baseline patient demographics and clinical characteristics in the safety analysis dataset

	*N* = 126
Age, years	
Mean ± SD	60.4 ± 17.7
Median	63.0
< 65, *n* (%)	67 (53.2)
≥ 65, *n* (%)	59 (46.8)
Sex, *n* (%)	
Male	68 (54.0)
Female	58 (46.0)
Body weight, kg	
Mean ± SD	55.2 ± 10.6
Median	55.8
ECOG PS, *n* (%)	
0–1	96 (76.2)
2–4	30 (23.8)
Baseline QTcF interval, ms	(*n* = 125)
Mean ± SD	417.7 ± 23.3
Median	421.0
LVEF, %	(*n* = 99)
Mean ± SD	64.2 ± 7.9
Median	64.0
Complications, *n* (%)	
Yes	73 (57.9)
History of cardiac, pulmonary, and kidney disease	30 (23.8)
Active double cancers other than AML	3 (2.4)
No	53 (42.1)
AML mode of onset, *n* (%)	
De novo	112 (88.9)
Secondary	14 (11.1)
Disease duration, days	(*n* = 123)
Mean ± SD	374.5 ± 444.2
Median	257.0
Relapsed or refractory, *n* (%)	
Relapsed	55 (43.7)
Refractory	71 (56.3)
CNS involvement, *n* (%)	
Yes	6 (4.8)
No	120 (95.2)
History of previous HSCT, *n* (%)	
Yes	37 (29.4)
No	88 (69.8)
Unknown	1 (0.8)
Time from previous HSCT to quizartinib start, days	
Mean ± SD	255.3 ± 200.7
Median	220.0
Number of previous HSCT, *n* (%)	
One	30 (81.1)
Two	7 (18.9)
Previous use of other FLT3 inhibitors, *n* (%)	
Yes	80 (63.5)
No	46 (36.5)
Reasons for discontinuation of other FLT3 inhibitors, *n* (%)	
ADR	24 (30.0)
Resistance	52 (65.0)
Unknown	4 (5.0)

ADR, adverse drug reaction; AML, acute myeloid leukemia; CNS, central nervous system; ECOG PS, Eastern Cooperative Oncology Group performance status; FLT3, FMS-like tyrosine kinase 3; HSCT, hematopoietic stem cell transplantation; LVEF, left ventricular ejection fraction; QTcF, QT interval corrected according to the Fridericia formula; SD, standard deviation

### Treatment

Six months after the start of quizartinib treatment, 22/126 patients (17.5%) in the safety analysis dataset continued to receive quizartinib and 104 (82.5%) discontinued the drug. The main reasons for treatment discontinuation were disease progression in 43 patients (34.1%), switching to HSCT in 34 (27.0%), and AEs in 23 (18.3%). The median overall treatment duration was 71.0 days.

The maximum daily dose of quizartinib during the treatment period was 17.7 mg in 15 patients (11.9%), 26.5 mg in 62 patients (49.2%), > 26.5 mg to < 53.0 mg in three patients (2.4%), and 53.0 mg in 46 patients (36.5%).

Forty patients (31.7%) received concomitant strong CYP3A inhibitors, the most common of which were voriconazole (*n* = 24; 19.0%) and itraconazole (*n* = 13; 10.3%). Among the 62 patients who received quizartinib at the maximum daily dose of 26.5 mg, 19 had coadministration with strong CYP3A inhibitors and 43 did not. Drugs known to cause QT interval prolongation were received by 65 patients (51.6%), the most common of which were fluconazole (*n* = 24; 19.0%), voriconazole (*n* = 24; 19.0%), and itraconazole (*n* = 13; 10.3%).

### Safety

TEAEs were reported in 85 patients (67.5%); 61 (48.4%) had a grade ≥ 3 TEAE, 35 (27.8%) had a serious TEAE, and 31 (24.6%) had a TEAE leading to discontinuation of quizartinib (Table 2).

**Table 2:** Summary of adverse events in the safety analysis dataset^a^

*n* (%)	*N* = 126
TEAEs	85 (67.5)
ADRs	68 (54.0)
Grade ≥ 3 TEAEs	61 (48.4)
Grade ≥ 3 ADRs	44 (34.9)
Serious TEAEs	35 (27.8)
Serious ADRs	20 (15.9)
TEAEs leading to discontinuation of quizartinib	31 (24.6)
ADRs leading to discontinuation of quizartinib	20 (15.9)

ADR, adverse drug reaction; TEAE, treatment-emergent adverse event

^a^The survey included all reported events, including those listed in the safety specifications

A total of 68 patients (54.0%) developed an ADR; 44 (34.9%) had a grade ≥ 3 ADR, 20 (15.9%) had a serious ADR, and 20 (15.9%) had an ADR leading to discontinuation of quizartinib (Table 2). The most common ADRs of any grade were neutrophil count decreased, platelet count decreased, QT interval prolongation, anemia, and white blood cell count decreased (Table 3). Grade ≥ 3 ADRs reported in ≥ 2 patients included neutrophil count decreased (*n* = 16; 12.7%), platelet count decreased (*n* = 14; 11.1%), white blood cell count decreased (*n* = 8; 6.3%), anemia (*n* = 7; 5.6%), febrile neutropenia (*n* = 4; 3.2%), and QT interval prolongation and hepatic function abnormal (*n* = 2 patients each; 1.6%). Serious ADRs that occurred in ≥ 2 patients were neutrophil count decreased and platelet count decreased (*n* = 6 patients each; 4.8%), anemia and white blood cell count decreased (*n* = 3 patients each; 2.4%), and febrile neutropenia (*n* = 2; 1.6%). ADRs leading to quizartinib discontinuation in ≥ 2 patients were neutrophil count decreased (*n* = 8; 6.3%), platelet count decreased (*n* = 5; 4.0%), and nausea (*n* = 3; 2.4%).

**Table 3:** Summary of adverse drug reactions in the safety analysis dataset (*N* = 126)

ADRs occurring in ≥ 2% of patients, n (%)	Any grade	Grade ≥ 3
Neutrophil count decreased	18 (14.3)	16 (12.7)
Platelet count decreased	18 (14.3)	14 (11.1)
QT interval prolongation^a^	17 (13.5)	2 (1.6)
Anemia	8 (6.3)	7 (5.6)
White blood cell count decreased	8 (6.3)	8 (6.3)
Febrile neutropenia	4 (3.2)	4 (3.2)
Hepatic function abnormal	4 (3.2)	2 (1.6)
Nausea	4 (3.2)	1 (0.8)

ADR, adverse drug reaction; ECG, electrocardiogram

^a^124/126 patients underwent post-baseline ECG

#### Safety specifications

**Fig. 2:**
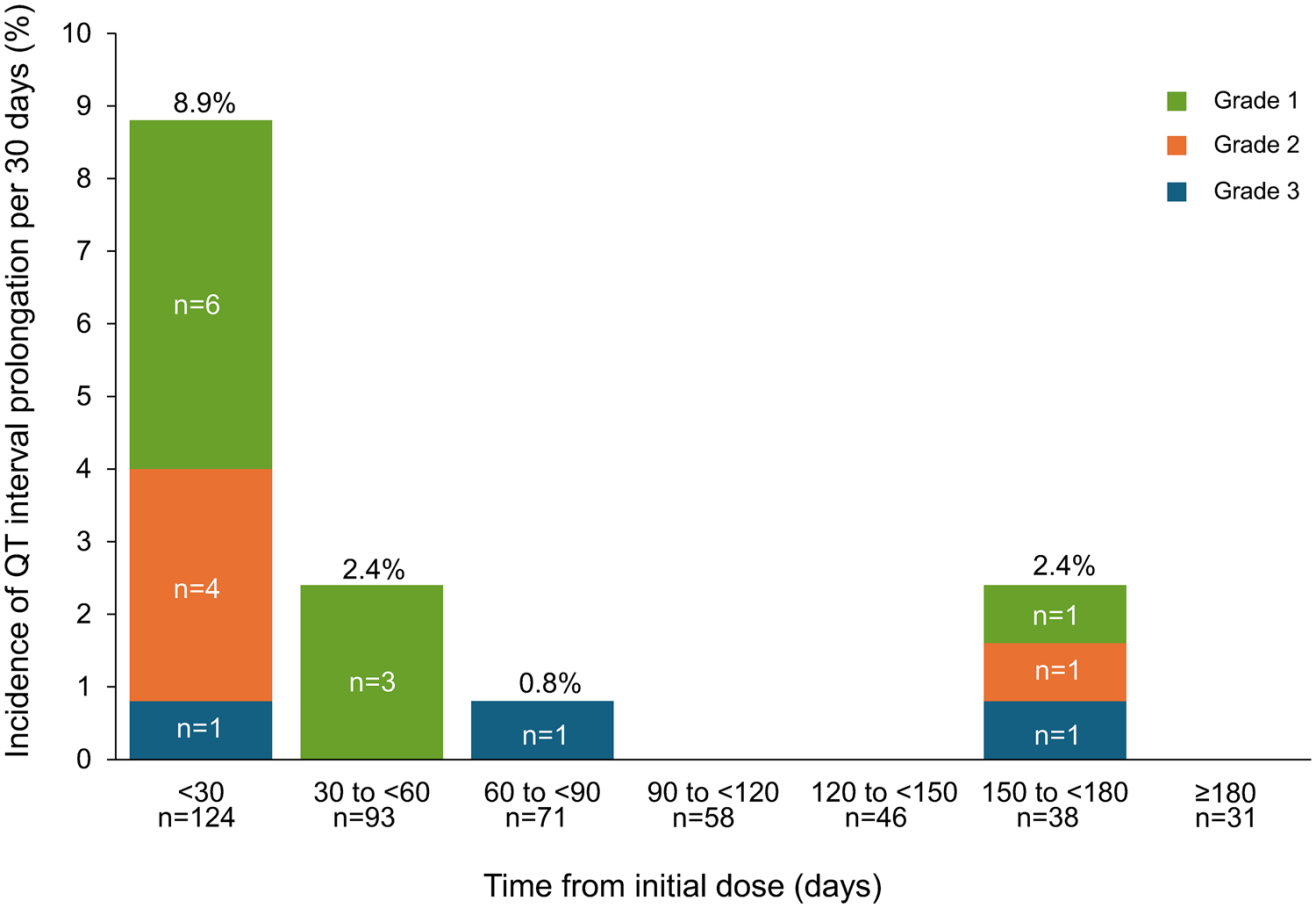
Incidence of QT interval prolongation by grade and time of initial onset. Patients who did not have a single post-quizartinib electrocardiogram were excluded from the safety analysis dataset

Among 124 patients who underwent post-baseline ECG, 18 (14.5%) experienced an ADR of QT interval prolongation. These events included ECG QT prolonged in 17 patients (13.7%) and long QT syndrome in one patient (0.8%). The severity of QT interval prolongation was mostly grade 1 or 2; grade 3 prolongation was less frequent (Fig. 2). No patients experienced clinical symptoms associated with QT interval prolongation (i.e., grade 4). The first QT interval prolongation event most commonly occurred < 30 days after quizartinib administration (8.9%), followed by 30– < 60 days, and 150– < 180 days (2.4% each; Fig. 2). One patient discontinued treatment due to a quizartinib treatment-related QT interval prolongation event. According to the attending physician, all 18 patients had recovered or were recovering. The mean duration from onset of QT interval prolongation to the date of recovery or outcome assessment in all 18 patients was 24.0 ± 27.7 days. Five patients experienced a total of six AEs of QT interval prolongation > 500 ms at 7, 27, 87, 133, 161, and 172 days from the start of treatment (Table 4). In one of these patients (Patient 4), QT prolongation occurred when quizartinib was re-administered following interruption for HSCT (on Days 133 and 161) and was associated with concomitant use of olanzapine or moxifloxacin. This patient also had a history of Wenckebach type AV block. All five patients recovered or were recovering after dose interruption/reduction within 15 days from the event onset. Of note, there were six patients whose QT interval prolongation exceeded 500 ms, but this event was not reported as an AE for one of these patients (Patient 6 in Table 4) because her ECG QT interval was > 500 ms at baseline and did not change.

**Table 4:** Characteristics of six patients^a^ with QT interval prolongation > 500 ms

	Patient 1	Patient 2	Patient 3	Patient 4^b^		Patient 5	Patient 6
Sex	Female	Female	Male	Male		Female	Female
Age, years	10 s	70 s	50 s	30 s		50 s	70 s
Baseline QTcF interval, ms	420.0	450.0	431.2	422.0		388.0	520.0
Maximum QTcF interval, ms	558.7	515.0	558.9	561.0		562.0	521.0
Quizartinib daily dose at onset, mg	26.5	26.5	17.7	26.5	17.7	17.7	NA
Time from start of treatment to onset, days	27	7	87	133	161	172	NA
Serious	No	No	No	Yes	No	No	NA
Treatment intervention	Interruption	Dose reduction	Interruption	Interruption	Interruption	Interruption	NA
Other measures	None	None	Unknown	None	Unknown	None	NA
Outcome	Recovering	Recovering	Recovered	Recovered	Recovered	Recovered	NA
Time from onset of AE to outcome, days	15	5	8	2	8	15	NA
Causality to quizartinib	Related	Related	Related	Related	Related	Related	NA
Cardiac disease history	None	None	None	Wenckebach type AV block		None	None
Concomitant medications^c^	Fluconazole	Unknown	Itraconazole	Itraconazole, voriconazole, posaconazole, moxifloxacin		Voriconazole, posaconazole	None
Possible causality to other factors	None	None	None	Olanzapine	Moxifloxacin	None	NA

^a^Includes details for 5 out of 6 patients who exceeded 500 ms, of which 5 were reported by the physician as AEs

^b^Patient 4 had two events

^c^Concomitant use of the following medications was investigated: strong CYP3A inhibitors; drugs known to prolong the QT interval

AE, adverse event; AV, atrioventricular; CYP, cytochrome P450; ECG, electrocardiogram; NA, not available; QTcF, QT interval corrected according to the Fridericia formula

No statistically significant differences were observed in the incidence of QT interval prolongation between patients receiving quizartinib with (16.9% [11/65]) versus without (11.9%[7/59]) concomitant use of drugs known to cause QT interval prolongation, or with (17.5% [7/40]) versus without (13.1% [11/84]) concomitant use of strong CYP3A inhibitors.

**Fig. 3:**
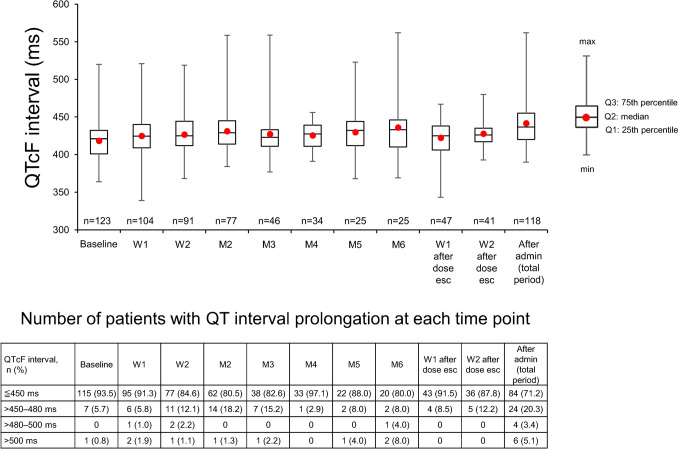
Box-and-whisker plot showing min, Q1, median, mean, Q3, max of QTcF interval over time. Red dot denotes mean. admin, administration; esc, escalation; M, month; max, maximum; min, minimum; Q1, 25th percentile; Q3, 75th percentile; QTcF, QT interval corrected according to the Fridericia formula; W, week

The median QTcF interval at baseline was 421.0 ms (Fig. 3). This extended after the start of treatment regardless of time point, with no clear time point at which QTcF interval extension was likely to occur.

Other safety specification events included four differentiation syndrome-like symptoms in three patients (2.4%; fever [*n* = 2], edema [*n* = 1], and rash [*n* = 1]), and acute kidney injury in one patient. No events related to myocardial infarction or interstitial lung disease were reported.

### Effectiveness

**Fig. 4:**
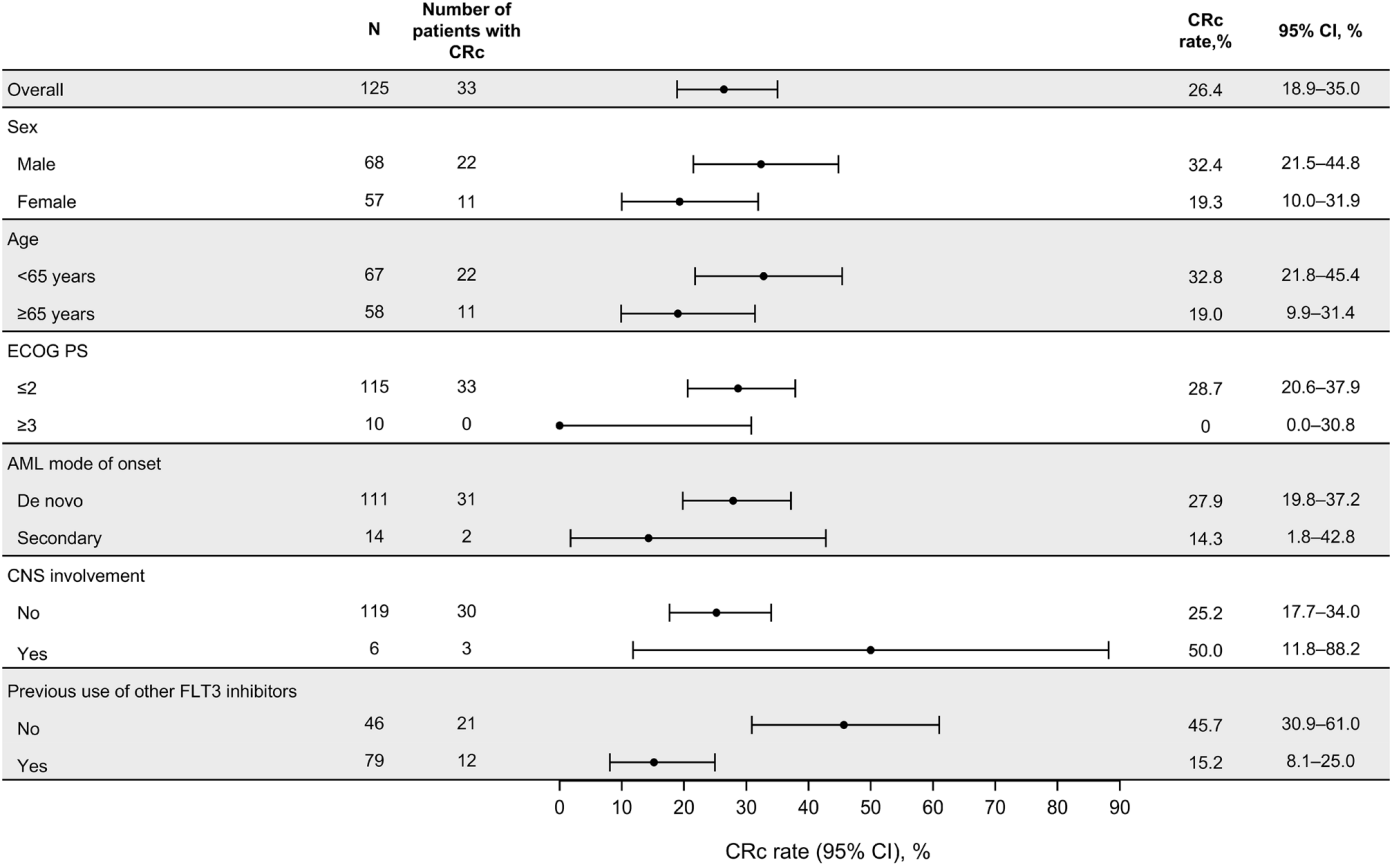
Composite complete remission rate by subgroup. Point estimates and 95% CIs for CRc rates are shown. AML, acute myeloid leukemia; CI, confidence interval; CNS, central nervous system; CRc, composite complete remission; ECOG PS, Eastern Cooperative Oncology Group performance status; FLT3, FMS-like tyrosine kinase 3

Six months after the start of treatment, the best antitumor response was CR in 17 patients (13.6%), CRp in five patients (4.0%), and CRi in 11 patients (8.8%), for a CRc rate of 26.4%. In subgroup analyses, the CRc rate was lower in patients with prior FLT3 inhibitor therapy (15.2%) than in those without prior FLT3 inhibitor therapy (45.7%; Fig. 4). In a post-hoc analysis, of 23 patients who discontinued other FLT3 inhibitors due to AEs, six (26.1%) achieved CRc, and of 52 patients who discontinued due to resistance to other FLT3 inhibitors, five (9.6%) achieved CRc. Post-dose bone marrow examinations were not performed in 61 of 125 patients (48.8%). Patients who had not been evaluated for tumor response were treated as non-responders. In a post-hoc analysis conducted in the 64 patients who did undergo bone marrow examinations, the CRc rate was 51.6%.

### Hematopoietic stem cell transplantation

In the 6 months after the start of quizartinib treatment, 35 patients (28.0%) underwent allogeneic HSCT without other AML therapy. Of these, CRc was achieved by 15 patients (45.5%) and not achieved by 20 patients (21.7%).

A total of 34 patients discontinued quizartinib because they transitioned to HSCT. In these patients, the mean ± SD duration from the start of quizartinib treatment to the day of HSCT was 68.0 ± 38.6 days and the median (range) was 55.5 (19–199) days. At 6 months, 15 patients who had discontinued quizartinib to undergo HSCT had restarted the drug. The mean ± SD duration between the date of HSCT treatment and the resumption of quizartinib administration was 53.1 ± 16.4 days and the median (range) was 51.0 (33–91) days.

## Discussion

The results of this PMS showed that the safety profile of quizartinib in routine clinical practice in Japanese patients with R/R *FLT3*-ITD-positive AML did not differ from the known safety profile of quizartinib, based on data from prescribing information [8, 10, 16] and clinical trials [6, 7]. ADRs occurred in about half (54.0%) of patients, the most common (all grades) being neutrophil count decreased, platelet count decreased, QT interval prolongation, anemia, and white blood cell count decreased.

Treatment with quizartinib is associated with an increased risk of QT interval prolongation [8, 10, 16]. In this PMS, ADRs associated with QT interval prolongation occurred in 14.5% of patients treated with quizartinib, and the first event typically occurred within 30 days of treatment initiation, but sometimes occurred after 150 days. This suggests that periodic QT monitoring by ECG should continue as part of routine clinical care during quizartinib treatment. Only six out of 124 patients had QT interval prolongation > 500 ms in this PMS. One of these patients (Patient 4) had a history of Wenckebach type AV block, but this was unlikely to be directly related to drug-induced QT prolongation due to differences in the underlying electrophysiological mechanisms. There were no QT interval prolongation grade ≥ 4 events or events associated with symptomatic arrhythmias, including torsade de pointes and ventricular arrhythmia. QT interval prolongation was effectively managed with existing risk minimization measures, such as dose interruption/reduction.

Differentiation syndrome-like symptoms were reported in three patients in this PMS. These patients were unlikely to have experienced typical differentiation syndrome associated with marked leukocytosis induced by quizartinib, as they did not show marked leukocytosis during the occurrence of the event; the differentiation syndrome-like symptoms may have been due to other etiologies such as complications or the primary disease (AML).

More than half of the patients (63.5%) had a history of treatment with another FLT3 inhibitor; of these, 65.0% had discontinued the FLT3 inhibitor due to resistance. The CRc rate observed in this PMS (26.4%) was determined by the attending physician based on bone marrow examinations that were judged to be necessary by the attending physician in clinical practice. Approximately half of the patients (48.8%) did not undergo a bone marrow test for effectiveness assessment and were categorized as non-responders. A possible reason for not undergoing bone marrow examination includes disease progression; however, the reasons for not undergoing testing were not collected. Lack of bone marrow examination likely resulted in underestimation of the effectiveness of quizartinib in this PMS. In the post-hoc analysis, the CRc rate in 64 patients who underwent effectiveness evaluation after quizartinib treatment was 51.6%. This CRc rate may be an overestimation because it excluded patients who did not undergo bone marrow testing after quizartinib administration. Additionally, subgroup analyses highlighted several factors that may affect the effectiveness of quizartinib treatment in this PMS; these include a history of FLT3 inhibitor use, an ECOG PS score of ≥ 3, or secondary AML, the latter two of which were exclusion criteria in the QuANTUM-R study. Despite the small sample size in the subgroup analysis, the pattern suggests that these known factors could have an impact on the effectiveness of the treatment.

Compared with participants in the QuANTUM-R study [6], those in this PMS were older (median age 63 vs 55 years), had more adverse prognostic factors (e.g., poor ECOG PS, secondary AML), and had greater prior exposure to FLT3 inhibitors. Many patients in this PMS switched to quizartinib due to resistance to previous FLT3 inhibitors; this prior exposure had a substantial impact on effectiveness (Fig. 4). Although quizartinib was more frequently administered in later treatment lines than in the QuANTUM-R study, its safety profile was not significantly different.

Importantly, differences in design and inclusion criteria between this PMS and QuANTUM-R make it challenging to compare results of the two studies. Further, it is difficult to determine the effectiveness of quizartinib based on the results of this PMS.

This PMS had several limitations. First, due to the inability to obtain consent for publication from about half of the patients, the number of patients included in this analysis was approximately half of the overall quizartinib treated population. However, comparison of patient demographics between the overall and consented populations, and between the consented and non-consented populations, revealed that there were no apparent differences that would substantially impact the interpretation of the results. Second, this PMS did not have a control group because it was designed as a single-arm descriptive analysis of real-world clinical practice. All of these factors may lead to inherent risks for bias. In addition, the PMS protocol did not specify the exact timing of response assessments, and almost half of patients (48.8%) had no response assessment. More than half of the patients (63.5%) had a history of treatment with another FLT3 inhibitor, and among them, 65.0% had discontinued the FLT3 inhibitor due to resistance. Finally, the minimum amount of information necessary to evaluate the safety and effectiveness of quizartinib was collected, and not all medical history and concomitant medications were investigated. Only medical history and concomitant medications that may affect the safety and effectiveness of quizartinib were investigated. All complications were collected, but medical history only included cardiac disease, pulmonary disease, and kidney disease. Only information on three types of concomitant medications were collected: strong CYP3A inhibitors; drugs known to prolong QT interval; and anticancer drugs used for AML other than quizartinib. It was difficult to determine the influence of QT-prolonging drugs on QT interval prolongation in this PMS due to the limited number of relevant patients.

## Conclusions

The results of this PMS suggest that the safety profile of quizartinib in patients with R/R *FLT3*-ITD-positive AML treated in routine clinical practice in Japan did not differ from the known safety profile of quizartinib, especially with regard to QT interval prolongation. No new safety concerns were identified. QT interval prolongation in patients treated with quizartinib can be managed with current risk minimization measures.

## Data Availability

The datasets used and/or analyzed during the current post-marketing surveillance and the individual patient data of this survey will not be shared.
